# Association between hypomagnesemia and serum lactate levels in patients with sepsis: a retrospective observational study

**DOI:** 10.1186/s44158-024-00158-2

**Published:** 2024-04-03

**Authors:** Ken Tonai, Shinshu Katayama, Kansuke Koyama, Hisashi Imahase, Shin Nunomiya

**Affiliations:** Division of Intensive Care, Department of Anesthesiology and Intensive Care Medicine, Jichi Medical University School of Medicine, Tochigi, Japan

**Keywords:** Lactate, Lactic acidosis, Magnesium, Magnesium deficiency, Sepsis

## Abstract

**Background:**

Sepsis-3 emphasizes the recognition of sepsis-induced cellular metabolic abnormalities, and utilizes serum lactate level as a biomarker of cellular metabolic abnormalities. Magnesium plays an important role as a cofactor in glucose metabolism, although it is not well known that magnesium deficiency causes elevated serum lactate levels. Additionally, it remains unclear how magnesium status affects the role of serum lactate levels as a marker of metabolic abnormalities in sepsis. Thus, this study aimed to investigate the association between serum magnesium and lactate levels in patients with sepsis and explore this relationship from the perspectives of time course and circulatory abnormalities.

**Methods:**

This retrospective observational study of adult patients with sepsis was performed at the 16-bed intensive care unit of Jichi Medical University Hospital between June 2011 and December 2017. The relationship between serum magnesium and lactate levels for 5 days from intensive care unit admission was investigated along the time course. Multivariate logistic regression analysis was performed to evaluate the association between serum magnesium and lactate levels during intensive care unit admission.

**Results:**

Among 759 patients included, 105 had hypomagnesemia (magnesium level < 1.6 mg/dL), 558 had normal serum magnesium levels (1.6–2.4 mg/dL), and 96 had hypermagnesemia (magnesium level > 2.4 mg/dL) at intensive care unit admission. From intensive care unit admission to day 5, the hypomagnesemia group had higher serum lactate levels and a higher frequency of lactic acidosis than the normal magnesium level and hypermagnesemia groups (70% vs. 51.6% vs. 50%; *P* < 0.001). Hypomagnesemia at intensive care unit admission was independently associated with lactic acidosis, i.e., lactic acid level > 2 mmol/L (odds ratio, 2.76; 95% confidence interval, 1.60–4.76; *P* < 0.001).

**Conclusions:**

Hypomagnesemia was associated with serum lactate levels in the early and post-resuscitation phases of sepsis. Further studies are needed to elucidate whether the magnesium status is associated with sepsis-induced cellular and metabolic abnormalities.

**Supplementary Information:**

The online version contains supplementary material available at 10.1186/s44158-024-00158-2.

## Background

Lactate is not just a metabolic product of anaerobic glycolysis, but also has important functions as an oxidative and gluconeogenic substrate and a signaling molecule [1, 2]. Lactate production is accelerated by hypermetabolic states such as sepsis [3]. When glycolysis causes an increase in pyruvate levels beyond the body’s capacity to metabolize it, the serum lactate level increases even in the absence of impaired oxygen transport [3, 4]. Additionally, lactate levels can provide prognostic information about patients with sepsis with and without hypotension [5] or impaired oxygen transport [6]. The European Society of Intensive Care Medicine and Society of Critical Care Medicine defined septic shock in the Third International Consensus Definitions for Sepsis and Septic Shock (Sepsis-3) as a subset of sepsis that includes cellular and metabolic abnormalities with a serum lactate level of > 2 mmol/L in addition to circulatory abnormalities with persisting hypotension [7]. Thus, an elevated lactate level has significance as a marker of metabolic abnormalities in sepsis.

Magnesium (Mg) acts as a cofactor in several essential biochemical enzymatic reactions (such as nucleic acid and protein synthesis) and has a role in immune modulation [8, 9]. Mg is also associated with glycolysis and the tricarboxylic acid (TCA) cycle in intracellular glucose metabolism [10, 11]. Mg plays a role as a cofactor in the conversion of thiamine to thiamine pyrophosphate (TPP), which binds pyruvate dehydrogenase and converts pyruvate to acetyl coenzyme A [12]. Thus, Mg deficiency alters the metabolism of pyruvate to acetyl coenzyme A, resulting in increased lactate production [11–14] (eFigure 1, Additional file 1).

Hypomagnesemia (HypoMg) has been frequently identified [15, 16] and is associated with increased mortality in critically ill patients [17]. In our previous study on the association between HypoMg and disseminated intravascular coagulation in patients with sepsis, we found that patients with HypoMg upon intensive care unit (ICU) admission had higher serum lactate levels and frequency of septic shock than those with normomagnesemia (NormoMg) [18]. Additionally, HypoMg on admission was associated with lactic acidosis [15] and septic shock [19] in critically ill patients, and Mg supplementation improved serum lactate clearance in patients with sepsis presenting with NormoMg and without hypotension [20]. If the Mg status is associated with serum lactate as a biomarker of sepsis-induced cellular metabolic abnormalities, then the unknown association between Mg status and serum lactate levels could mislead physicians in the diagnosis and treatment of sepsis. However, no previous study has examined the relationship between serum magnesium and lactate levels in sepsis after the day of ICU admission. Thus, the manner in which serum Mg levels affect the pathophysiology of lactic acidosis in sepsis, including time phase and patient population, remains unclear.

To analyze in detail the findings of our previous study [18] in detail, the present was designed to retrospectively investigate the relationship between serum Mg and lactate levels from ICU admission to day 5 in patients with sepsis and additionally explore these relationships from the perspectives of time course, lactate clearance, and circulatory abnormalities.

## Methods

### Study design and data collection

This was a single-center, retrospective, and observational study of adult patients with sepsis in the 16-bed ICU of Jichi Medical University Hospital, Tochigi, Japan, between June 2011 and December 2017. In our previous study, we retrospectively assembled data from electronic medical records of adult patients (≥ 20 years) who met the Sepsis-3 definition [7] and were admitted to the ICU and created a dataset to analyze the association between HypoMg and coagulopathy [18]. The same dataset was used in this study. We excluded patients who were readmitted to the ICU and those whose data on serum Mg or lactate levels on the day of ICU admission were unavailable for analysis. This study was approved by the Jichi Medical University Hospital Bioethics Committee for Clinical Research (approval number: 21–034, date of approval: October 1, 2021). The procedures followed were in accordance with the ethical standards of the committee on human experimentation and Helsinki Declaration of 1975. The institutional ethics committee waived the need for informed consent due to the retrospective nature of the study.

### Lactic acidosis, lactate clearance, septic shock, and serum Mg level

Lactic acidosis was defined according to the criteria for septic shock in Sepsis-3 and in previous studies as follows: lactic acidosis (serum lactate level > 2 mmol/L) and severe lactic acidosis (serum lactate level > 4 mmol/L) [15, 21, 22]. Lactate clearance is the percentage reduction in lactate levels from ICU admission to the time of measurement. Lactate clearance on measurement was calculated using the following formula: (lactate level at ICU admission-lactate on measurement) × 100/lactate level at ICU admission (%) [22]. According to Sepsis-3, septic shock was clinically defined as sepsis with persisting hypotension, requiring vasopressors to maintain an MAP of ≥ 65 mmHg and a serum lactate level > 2 mmol/L (18 mg/dL) despite adequate volume resuscitation [7].

The frequency of lactic acidosis according to the deciles of the serum Mg level at ICU admission demonstrated a J-shaped curve (eFigure 2, Additional file 1). Thus, we categorized the serum Mg level into three groups using the definition in other studies on Mg [15, 23, 24]: HypoMg (Mg level < 1.6 mg/dL), NormoMg (1.6–2.4 mg/dL), and HyperMg (Mg level > 2.4 mg/dL). Similar to our previous study [18], based on the presence of HypoMg on days 2 and 3, we categorized patients with HypoMg on the day of ICU admission into two groups: persistent HypoMg (Mg level < 1.6 mg/dL) and resolved HypoMg (Mg level ≥ 1.6 mg/dL). Additionally, patients without HypoMg at ICU admission were divided into two groups: developed HypoMg (Mg level < 1.6 mg/dL) and non-HypoMg (Mg level ≥ 1.6 mg/dL).

### Statistical analyses

We present the data as medians with interquartile ranges for continuous variables and as counts with percentages for categorical variables. We compared the data in two or three groups according to serum Mg levels using the Mann–Whitney *U* and Kruskal–Wallis tests for continuous variables and the chi-square and Fisher’s exact test for categorical variables. When there were statistically significant differences among the three groups according to serum Mg levels, the Steel–Dwass test, chi-square test, or Fisher’s exact test with the Bonferroni correction was performed to determine significance in the setting of multiple comparisons. To examine the association between serum Mg and lactate levels with and without hypotension, subgroup analyses were performed according to the presence or absence of the need for vasopressors to maintain an MAP of ≥ 65 mmHg. Univariate and multivariate logistic regression analyses were performed to determine independent associations between serum Mg levels and lactic acidosis in complete case analyses. From amongst the variables extracted from our previous study [18], age, chronic comorbidities such as chronic live disease, blood pressure, vasopressor use, respiratory dysfunction, serum creatinine, platelet count, prothrombin time, and severity were considered to be potentially related to lactic acidosis in previous studies [15, 25–27]. We included age, chronic comorbidities, blood pressure, respiratory dysfunction, and serum creatinine as a single summary score, viz. the Acute Physiology and Chronic Health Evaluation (APACHE) II score rather than individual variables. Moreover, we added sex; an inflammatory biomarker, viz. C-reactive protein level; and an electrolyte, viz. ionized calcium level as covariates in the multivariate logistic regression models. In model 1, no adjustments were made. Model 2 was adjusted for sex and the APACHE II score. Model 3 was additionally adjusted for the need for vasopressors to maintain an MAP of ≥ 65 mmHg, C-reactive protein level, ionized calcium level, platelet count, and prothrombin time-international normalized ratio. All data were analyzed using JMP pro (version 16. 1. 0) software (SAS Institute Inc., Cary, NC, USA) and R package (version 4.0.4, R Foundation for Statistical Computing, Vienna, Austria). Statistical significance was set at *P* < 0.05.

## Results

### Patient characteristics

**Fig. 1:**
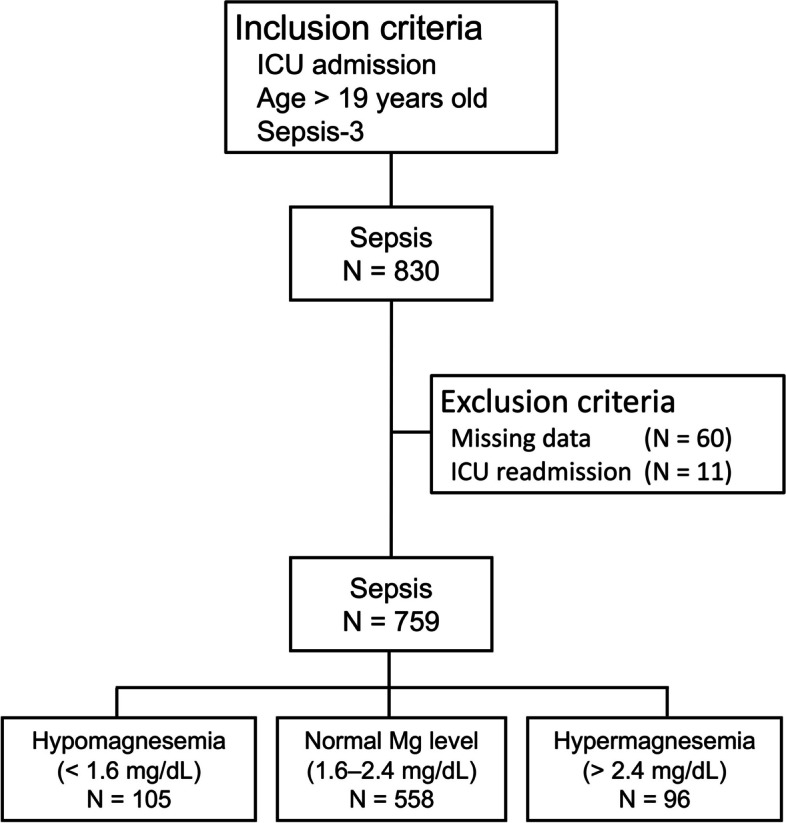
Flow chart of participant enrollment

During the study period, 830 patients with sepsis were admitted to the ICU. In total, 71 patients were excluded from this study—60 with missing data at the time of ICU admission and 11 who were readmitted to the ICU. Therefore, based on serum Mg levels on ICU admission, i.e., day 1, 759 patients were eligible for data analysis, with 105, 558, and 96 patients having HypoMg, NormoMg, and hypermagnesemia (HyperMg), respectively (Fig. 1). Table 1 shows the baseline characteristics of the 759 patients categorized into the HypoMg, NormoMg, and HyperMg groups. Compared with the NormoMg and HyperMg groups, the HypoMg group had higher serum lactate levels (median: 3.9 vs. 2.1, *P* < 0.01; 3.9 vs. 2.1, *P* < 0.01, respectively) and more patients with septic shock (70.5% vs. 40.1%., *P* < 0.01; 70.5% vs. 42.7%., *P* < 0.01, respectively). However, there were no differences in the frequency of the need for vasopressors to maintain a mean arterial pressure (MAP) of ≥ 65 mmHg on the ICU admission day among the three groups. The HypoMg group had a higher disease severity (median Acute Physiology and Chronic Health Evaluation [APACHE] II score: 25 vs. 23; *P* < 0.01) and hospital mortality (25.7% vs. 14.3%; *P* < 0.05) than the NormoMg group. However, there were no differences in disease severity and hospital mortality between the HypoMg and HyperMg groups.

**Table 1:** Characteristics of the study population

	HypoMg (Mg level: < 1.6 mg/dL) *N* = 105	NormoMg (1.6–2.4 mg/dL) *N* = 558	HyperMg (Mg level: > 2.4 mg/dL) *N* = 96	*P*-value
Age, years, median (IQR)	65 (53–74)	69 (59–78) *	70 (63–78) *	0.023
Male sex, n (%)	53 (50.5)	316 (56.6)	51 (53.1)	0.46
Body mass index (kg/m^2^)	23.0 (20.2–25.4)	22.0 (19.5–25.6)	23.3 (19.5–25.6)	0.20
Medical history, n (%)				
CKD with dialysis	2 (1.9)	43 (7.8)	11 (11.5) *	0.030
Chronic liver disease	15 (14.3)	47 (8.5)	8 (8.3)	0.16
Diabetes mellitus	21 (20.0)	158 (28.4)	32 (33.3)	0.094
Hypertension	41 (39.1)	283 (50.9)	57 (59.4) *	0.014
Origin of sepsis, n (%)				0.12
Abdomen	46 (43.8)	310 (55.6)	41 (42.7)	
Thorax	24 (22.9)	114 (20.4)	25 (26.0)	
Urinary tract	6 (5.7)	24 (4.3)	4 (4.2)	
Other	29 (27.6)	110 (19.7)	26 (27.1)	
Laboratory values, median (IQR)				
Mg, mg/dL	1.4 (1.3–1.5)	2.0 (1.8–2.2) **	2.7 (2.5–2.9) ** ††	< 0.001
Albumin, mg/dL	2.4 (1.9–2.9)	2.4 (2.0–2.8)	2.3 (2.1–2.8)	0.81
Bilirubin, mg/dL	0.92 (0.64–1.57)	0.87 (0.59–1.40)	0.72 (0.50–1.29) * †	0.048
Creatinine, mg/dL	1.51 (0.83–2.20)	1.00 (0.67–1.96) *	1.72 (0.93–3.82) ††	< 0.001
CRP, mg/dL	9.5 (5.0–16.0)	13.7 (5.9–23.5) *	13.4 (7.7–21.1) *	0.019
Ionized calcium, mmol/L	0.99 (0.92–1.07)	1.06 (1.0–1.11) **	1.06 (0.98–1.16) **	< 0.001
Platelet, 10^4^/μL	11.2 (5.5–16.6)	14.8 (9.6–22.8) **	15.1 (10.0–19.9) **	< 0.001
PT-INR	1.52 (1.31–1.82)	1.37 (1.23–1.55) **	1.36 (1.23–1.61)	< 0.001
Organ support, n (%)				
Mechanical ventilation	91 (86.7)	457 (82.0)	81 (84.4)	0.45
Renal replacement therapy	34 (32.4)	113 (20.3) *	35 (36.5) ††	< 0.001
Severity of disease				
SOFA score on ICU admission, median (IQR)	9 (6–11)	7 (4–9) *	9 (6–11) †	< 0.001
APACHE II score, median (IQR)	25 (20–33)	23 (17–29) **	27 (20–33) ††	< 0.001
Septic shock, n (%)	74 (70.5)	224 (40.1) **	41 (42.7) **	< 0.001
Need for vasopressors within ICU admission day, n (%)	86 (81.9)	398 (71.3)	74 (77.1)	0.055
Hospital mortality, n (%)	27 (25.7)	80 (14.3) *	24 (25.0) †	0.002

Continuous and categorical data are presented as medians with interquartile ranges (25th and 75th percentiles) and counts with percentages, respectively

Multiple comparisons were performed using the Steel–Dwass test, chi-square test, or Fisher's exact test with the Bonferroni correction

*Abbreviations*: *HypoMg* hypomagnesemia, *NormoMg* normomagnesemia, *HyperMg* hypermagnesemia, *Mg* magnesium, *IQR* interquartile range (first quartile to third quartile), *CKD* chronic kidney disease, *CRP* C-reactive protein, *PT-INR* prothrombin time-international normalized ratio, *SOFA* Sequential Organ Failure Assessment, *APACHE* Acute Physiology and Chronic Health Evaluation, *ICU* intensive care unit

Comparison with HypoMg: ^*^ *P* < 0.05; ^**^ *P* < 0.01. Comparison with NormoMg: ^†^ *P* < 0.05; ^††^ *P* < 0.01

### Comparison of serum lactate levels according to serum Mg levels from ICU admission to day 5

Although 759 patients were included in the study, the serum lactate and Mg levels until day 5 were not available for 215 patients, among whom 32 died, 159 were discharged from the ICU, and 24 had missing data. The serum lactate level and frequency of lactic acidosis decreased gradually from admission to day 4. The HypoMg group had higher serum lactate levels and more patients with lactic acidosis than the NormoMg group from admission to day 5 and HyperMg group from admission to day 2 (Table 2).

**Table 2:** Comparison of serum lactate parameters by serum magnesium levels from ICU admission to day 5

	HypoMg (Mg level < 1.6 mg/dL)	NormoMg (1.6–2.4 mg/dL)	HyperMg (Mg level > 2.4 mg/dL)	*P*-value
ICU admission	*N* = 105	*N* = 558	*N* = 96	
Lactate level, mmol/L	3.9 (2.2–6.1)	2.1 (1.3–3.3) **	2.1 (1.4–3.6) **	< 0.001
Lactic acidosis n (%)	84 (70)	288 (51.6) **	48 (50) **	< 0.001
Severe lactic acidosis n (%)	48 (45.7)	103 (18.5) **	23 (24) **	< 0.001
Day 2	*N* = 105	*N* = 553	*N* = 65	
Lactate level, mmol/L	2.3 (1.5–3.6)	1.4 (1.0–2.2) **	1.6 (1.2–2.1) **	< 0.001
Lactic acidosis n (%)	61 (58.1)	152 (27.5) **	16 (24.6) **	< 0.001
Severe lactic acidosis n (%)	21 (20)	42 (7.6) **	4 (6.2) *	< 0.001
Day 3	*N* = 63	*N* = 560	*N* = 56	
Lactate level, mmol/L	1.8 (1.2–2.3)	1.3 (1.0–1.9) **	1.5 (1.1–1.9)	0.001
Lactic acidosis n (%)	22 (34.9)	109 (19.5) *	9 (16.1)	0.011
Severe lactic acidosis n (%)	8 (12.7)	20 (3.6) **	0 (0) *	< 0.001
Day 4	*N* = 42	*N* = 516	*N* = 50	
Lactate level, mmol/L	1.6 (1.1–2.3)	1.3 (1.0–1.7) *	1.3 (1.0–1.7)	0.019
Lactic acidosis n (%)	13 (31.0)	84 (16.3)	4 (8.0) *	0.011
Severe lactic acidosis n (%)	5 (11.9)	11 (2.1) **	0 (0)	< 0.001
Day 5	*N* = 30	*N* = 472	*N* = 42	
Lactate level, mmol/L	1.8 (1.3–2.2)	1.3 (1.0–1.7) *	1.4 (1.0–1.9)	0.004
Lactic acidosis n (%)	11 (36.7)	70 (14.8) *	6 (14.3)	0.006
Severe lactic acidosis n (%)	2 (6.7)	8 (1.7)	0 (0)	0.13

Continuous and categorical data are presented as medians with interquartile ranges (25th and 75th percentiles) and counts with percentages, respectively

Multiple comparisons were performed using the Steel–Dwass test, chi-square test, or Fisher's exact test with the Bonferroni correction

*Abbreviations*: *HypoMg* hypomagnesemia, *NormoMg* normomagnesemia, *HyperMg* Hypermagnesemia, *Mg* magnesium, *ICU* intensive care unit

Comparison with HypoMg: * *P* < 0.05; ** *P* < 0.01. Comparison with NormoMg: ^†^ *P* < 0.05; ^††^ *P* < 0.01

### Comparison of serum lactate parameters between the persistent and resolved HypoMg groups

In total, 105 patients with HypoMg at ICU admission were divided into the persistent HypoMg group (on day 2, *N* = 68; on day 3, *N* = 35) and resolved HypoMg group (on day 2, *N* = 33; on day 3, *N* = 64) according to their serum Mg levels on days 2 and 3. There was no difference in the serum lactate level or frequency of lactic acidosis between the groups on days 2 and 3. The resolved HypoMg group had higher lactic clearance than the persistent HypoMg group on day 3 (51.9% vs. 34.6%, *P* = 0.027) (Table 3).

**Table 3:** Comparison of serum lactate parameters between the persistent and resolved hypomagnesemia groups

Day 2	Persistent HypoMg *N* = 68	Resolved HypoMg *N* = 33	*P*-value
Lactate level on ICU admission, mmol/L	4.1 (2.0–6.4)	3.6 (2.7–5.4)	0.49
Lactate level on day 2, mmol/L	2.5 (1.5–3.7)	2.0 (1.3–3.3)	0.21
Lactic acidosis on day 2, %	41 (60.1)	15 (45.5)	0.16
Severe lactic acidosis on day 2, %	15 (22.1)	4 (12.1)	0.23
Lactate clearance, %	23.5 (0–54.1)	47.6 (17.2–54.0)	0.12
Day 3	Persistent HypoMg *N* = 35	Resolved HypoMg *N* = 64	*P*-value
Lactate level on ICU admission, mmol/L	3.8 (1.9–5.4)	3.8 (2.7–6.2)	0.20
Lactate level on day 3, mmol/L	1.8 (1.3–2.3)	1.8 (1.2–2.4)	0.99
Lactic acidosis on day 3, %	12 (34.1)	23 (35.9)	0.87
Severe lactic acidosis on day 3, %	4 (11.4)	6 (9.4)	0.75
Lactate clearance, %	34.6 (9.1–57.1)	51.9 (29.1–69.1)	0.027

Continuous and categorical data are presented as medians with interquartile ranges (25th and 75th percentiles) and counts with percentages, respectively

*Abbreviations: HypoMg* hypomagnesemia, *ICU* intensive care unit

### Comparison of serum lactate parameters between the developed and non- HypoMg groups

In total, 654 patients without HypoMg at ICU admission were divided into the developed HypoMg group (on day 2, *N* = 37; on day 3, *N* = 28) and non-HypoMg group (on day 2, *N* = 586; on day 3, *N* = 553) according to their serum Mg levels on days 2 and 3. The group that developed HypoMg on day 2 or 3 had higher serum lactate levels, frequency of lactic acidosis, and serum lactate levels at ICU admission before developing HypoMg than the non-HypoMg group (Table 4).

**Table 4:** Comparison of serum lactate parameters between the developed hypomagnesemia and non-hypomagnesemia groups

Day 2	Developed HypoMg *N* = 37	Non-HypoMg *N* = 586	*P*-value
Lactate level on ICU admission, mmol/L	3.7 (2.4–5.8)	2.0 (1.3–3.1)	< 0.001
Lactate level on day 2, mmol/L	2.1 (1.1–3.5)	1.4 (1.0–2.1)	0.003
Lactic acidosis on day 2, %	20 (54.1)	154 (26.3)	< 0.001
Severe lactic acidosis on day 2, %	6 (16.2)	43 (7.3)	0.052
Lactate clearance, %	28.9 (8.2–55.3)	21.4 (0–44)	0.07
Day 3	Developed HypoMg *N* = 28	Non-HypoMg *N* = 553	*P*-value
Lactate level on ICU admission, mmol/L	3.0 (2.2–4.0)	2.0 (1.3–3.2)	0.007
Lactate level on day 3, mmol/L	1.7 (1.2–2.6)	1.3 (1.0–1.8)	0.012
Lactic acidosis on day 3, %	10 (35.7)	95 (17.2)	0.013
Severe lactic acidosis on day 3, %	4 (14.3)	14 (2.5)	< 0.001
Lactate clearance, %	34.7 (17.5–58.5)	34.5 (8.3–56.8)	0.61

Continuous and categorical data are presented as medians with interquartile ranges (25th and 75th percentiles) and counts with percentages, respectively

*Abbreviations*: *HypoMg* hypomagnesemia *ICU* intensive care unit

### Subgroup analysis

The group that required vasopressors to maintain an MAP of ≥ 65 mmHg had higher lactate levels, disease severity, and mortality (eTable 1, Additional file 1) than the group that did not. Patients with HypoMg had higher lactate levels and more frequent lactic acidosis than those with NormoMg and HyperMg in the subgroup that required vasopressors to maintain an MAP of ≥ 65 mmHg but not in the subgroup that did not require vasopressors (eTables 2 and 3, Additional files 1).

### Logistic regression analysis of factors associated with lactic acidosis

In the unadjusted analysis (model 1), the HypoMg group had increased odds of developing lactic acidosis (odds ratio [OR], 3.75; 95% confidence interval [CI], 2.26–6.22; *P* < 0.001) compared with the NormoMg group. This relationship remained highly significant after adjusting for disease severity and the need for vasopressors to maintain an MAP of ≥ 65 mmHg in models 2 and 3 (Table 5).

**Table 5:** Logistic regression analyses of factors associated with lactic acidosis

Magnesium	OR	95% CI	*P*-value
Model 1 (*N* = 759)			
NormoMg	Reference		
HypoMg	3.75	2.26–6.22	< 0.001
HyperMg	0.94	0.61–1.45	0.77
Model 2 (*N* = 759)			
NormoMg	Reference		
HypoMg	3.33	1.98–5.60	< 0.001
HyperMg	0.73	0.46–1.15	0.17
Model 3 (*N* = 755)			
NormoMg	Reference		
HypoMg	2.76	1.60–4.76	< 0.001
HyperMg	0.71	0.44–1.15	0.16

Data are presented as odds ratios (95% confidence intervals). Model 1: unadjusted magnesium levels. Model 2: adjusted for sex and APACHE II score. Model 3: adjusted for sex, APACHE II score, need for vasopressors to maintain a mean arterial pressure of ≥ 65 mmHg on the intensive care unit admission day, C-reactive protein level, ionized calcium level, platelet count, and prothrombin time-international normalized ratio

*Abbreviations*: *OR* odds ratio, *CI* confidence interval, *HypoMg* hypomagnesemia, *NormoMg* normomagnesemia, *HyperMg* Hypermagnesemia, *APACHE* Acute Physiology and Chronic Health Evaluation

## Discussion

Our retrospective observational study aimed to determine whether serum Mg levels were associated with serum lactate levels in patients with sepsis and if so, in which phase and in which specific population. The HypoMg group had high serum lactate levels and included many patients with lactic acidosis from ICU admission to day 5, and the patients who newly developed HypoMg on days 2 and 3 had high serum lactate levels. Subgroup analysis showed that HypoMg was associated with increased serum lactate levels in patients who required vasopressors to maintain an MAP of ≥ 65 mmHg but not in those who did not. These findings indicate that HypoMg is associated with lactic acid metabolism from the early resuscitation to the post-resuscitation phase of sepsis and in hemodynamically unstable patients with sepsis.

Serum lactate levels indicate the balance between the production and consumption of lactate [4]. The former involves glycolysis, and the latter involves the TCA cycle [2]. Accelerated glycolysis produces lactate according to the energy demands of various organs for biological reactions, and this phenomenon is known as sepsis; subsequently, lactate is used as an energy substrate via the TCA cycle for oxidative phosphorylation or gluconeogenesis [1]. As a cofactor, Mg is principally related to various enzymatic activities of glucose metabolism in both glycolysis and the citric acid cycle [11]. Among the various enzymatic reactions, Mg is essential for the activation of pyruvate dehydrogenase, which converts pyruvate to acetyl coenzyme A, via TPP [3, 11, 12]. Although some studies have reported that thiamine may improve the prognosis of sepsis in patients with thiamine deficiency [28], it has not been clearly shown to improve prognosis in sepsis [29]. However, studies have reported that thiamine deficiency is potentially involved in lactic acidosis in patients with sepsis with thiamine deficiency [30, 31]. If pyruvate dehydrogenase activity is impaired, increased glycolysis, especially during sepsis, can easily exceed the capacity of mitochondrial respiration to metabolize pyruvate [3, 4]. Thus, Mg deficiency could impair pyruvate dehydrogenase activity, resulting in elevated lactate levels.

When investigating the association between Mg and lactate, it is important to distinguish between increased lactate levels in the early resuscitation and post-resuscitation phases because hyperlactatemia has different clinical implications in the two phases [3]. Inadequate tissue oxygenation is one of the factors contributing to elevated serum lactate levels, and hemodynamic resuscitation reduces lactate levels in the early resuscitation phase of sepsis. Magnesium has also been implicated in the cardiac conduction as an essential cofactor of the Na–K-ATP pump, and Mg status therefore influences myocardial excitability [10, 32]. Magnesium deficiency is a risk for arrhythmias such as atrial fibrillation [9, 10, 32], which may result in hemodynamical instability and elevated serum lactate levels. However, persistently elevated serum lactate levels in the post-resuscitation phase often occur independently of whole-body oxygen delivery; therefore, causes of hyperlactatemia other than inadequate whole-body oxygen delivery must be considered [3, 4]. Our findings demonstrated that HypoMg was associated more strongly with high lactate levels than NormoMg even in the post-resuscitation phase when the lactate levels decreased (days 2–5). This result may support the hypothesis that the theoretical mechanism underlying the association between HypoMg and increased lactate level is impaired pyruvate dehydrogenase and not inadequate oxygen delivery.

Mild hyperlactatemia in hemodynamically stable patients with sepsis is due to impaired lactate clearance rather than lactate overproduction [33]. We also showed that hypomagnesemia was associated with serum lactate levels in the subgroup that required vasopressors to maintain an MAP of ≥ 65 mmHg but not in the subgroup that did not require vasopressors. However, the sample size of the subgroup that did not require vasopressors might be too small for evaluation. The subgroup that did not require vasopressors to maintain an MAP of ≥ 65 mmHg had lower serum lactate levels (median: 1.6 mmol/L) and less frequent lactic acidosis (31.3%) than the subgroup that required vasopressors. These findings suggest that Mg is relevant to lactate production rather than lactate clearance. Thus, in situations of increased glycolytic flux during sepsis, HypoMg may reduce mitochondrial oxidative capacity, causing hyperlactatemia.

Intracellular Mg is associated with glucose metabolism but not with serum Mg levels. Although hypomagnesemia is not a good indicator of Mg deficiency [34], Mg deficiency is usually detected due to refractory HypoMg because there is no simple or rapid test to measure intracellular and total body Mg levels [8]. Our study demonstrated that HypoMg and its development are associated with increased serum lactate levels. Interestingly, the developed HypoMg group already had significantly higher serum lactate levels before onset than the non- HypoMg group. Compared with the persistent HypoMg group, the resolved HypoMg group did not show significant improvements in lactic acidosis, even with improvement in HypoMg. These findings indicate that the developed or resolved HypoMg group may have occult intracellular Mg deficiency. To assess the relationship between Mg deficiency and various metabolic reactions, a simple method for measuring intracellular or total body Mg levels must be established. Further studies are required to clarify the mechanism underlying the relationship between Mg and lactate levels and whether treatment of HypoMg may be a potential therapeutic strategy for hyperlactatemia.

Our study had some limitations. First, this was a single-center, retrospective, and observational study. Because of the retrospective nature of this study, data on serum lactate and Mg levels from admission to day 5 in approximately 30% of the included patients were not available due to death or ICU discharge; the number of patients with missing data among the 759 patients included in this study was as follows: 36 (4.7%) on day 2, 80 (10.5%) on day 3, 147 (19.4%) on day 4, and 213 (28.0%) on day 5. Hence, selection bias could have occurred. Due to the large number of patients with missing data on days 4 and 5, we investigated serum lactate only for each change in serum Mg status from ICU admission to days 2 and 3. Second, we did not investigate the baseline Mg profile before sepsis (Mg deficiency or HypoMg), nutritional status (reduced intake or Mg supplementation), cause of Mg deficiency or HypoMg such as comorbidities (gastrointestinal and renal loss) and drug usage (diuretics and proton-pump inhibitor) [32]. Third, there was a lack of information on factors that may affect serum Mg levels during ICU stay, such as Mg administration, diuretics, and dialysis. Fourth, we did not evaluate the mechanism underlying the association between Mg and lactate because we did not investigate glycolysis and oxidative phosphorylation of intracellular metabolic processes through thiamine-dependent reactions.

## Conclusion

We demonstrated that HypoMg was associated with lactic acidosis during the early and post-resuscitation phases of sepsis, especially in hemodynamically unstable patients with sepsis. However, it is unknown whether Mg status is associated with the pathophysiology of sepsis-induced cellular and metabolic abnormalities. Further studies are needed to elucidate the mechanism underlying the relationship between Mg status and lactate levels in sepsis and whether Mg is associated with sepsis-induced cellular and metabolic abnormalities.

### Supplementary Information

**Supplementary Material 1.**

## Data Availability

The datasets generated and/or analyzed during the current study are not publicly available because of patient-related confidentiality.

## Abbreviations

ICU: Intensive care unit
Mg: Magnesium
TCA: Tricarboxylic acid cycle
TPP: Thiamine pyrophosphate
MAP: Mean arterial pressure
APACHE: Acute Physiology and Chronic Health Evaluation
HypoMg: Hypomagnesemia
NormoMg: Normomagnesemia
HyperMg: Hypermagnesemia
