# Supplementary material for: Association between hypomagnesemia and serum lactate levels in patients with sepsis: a retrospective observational study
Source: J Anesth Analg Crit Care. 2024 Apr 3;4:23. doi: 10.1186/s44158-024-00158-2 (PMC10988873; doi:10.1186/s44158-024-00158-2)
Supplement: Supplementary file 1 — Supplementary Material 1. [file 44158_2024_158_MOESM1_ESM.docx]

**ONLINE SUPPLEMENT**

**Title:** Association between hypomagnesemia and serum lactate in sepsis: A retrospective observational study

**Authors:** Ken Tonai, Shinshu Katayama, Kansuke Koyama, Hisashi Imahase, and Shin Nunomiya

**Table of Contents**

**eFiugre 1.** The role of magnesium in the conversion of pyruvate to acetyl coenzyme A.

**eFigure 2.** The frequency of lactic acidosis according to decile of serum magnesium concentration.

**eTable 1.** Comparison between Need for vasopressors and non-vasopressors.

**eTable 2.** Comparison of serum lactate parameters according to serum magnesium levels on ICU admission in subgroup of need for vasopressor.

**eTable 3.** Comparison of serum lactate parameters according to serum magnesium levels on ICU admission in subgroup of no need for vasopressor.

**eFiugre 1. The role of magnesium in the conversion of pyruvate to acetyl coenzyme A.**

**
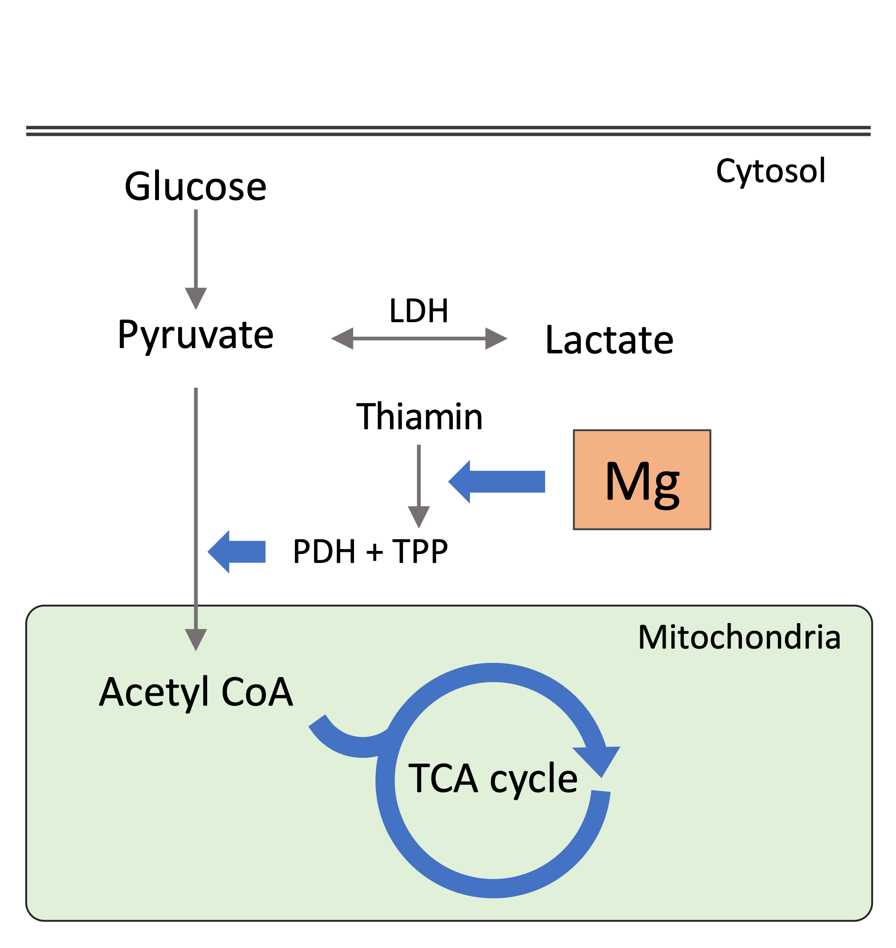
**

Magnesium plays a role in the conversion of thiamine by thiamine pyrophosphokinase to the active form thiamine pyrophosphate (TPP), a cofactor of the pyruvate dehydrogenase reaction that converts pyruvate to acetyl coenzyme A. Abbreviations: Mg, magnesium; TPP, thiamine pyrophosphate; PDH, pyruvate dehydrogenase; TCA, tricarboxylic acid.

**eFigure 2. The frequency of lactic acidosis according to decile of serum magnesium concentration.**
**
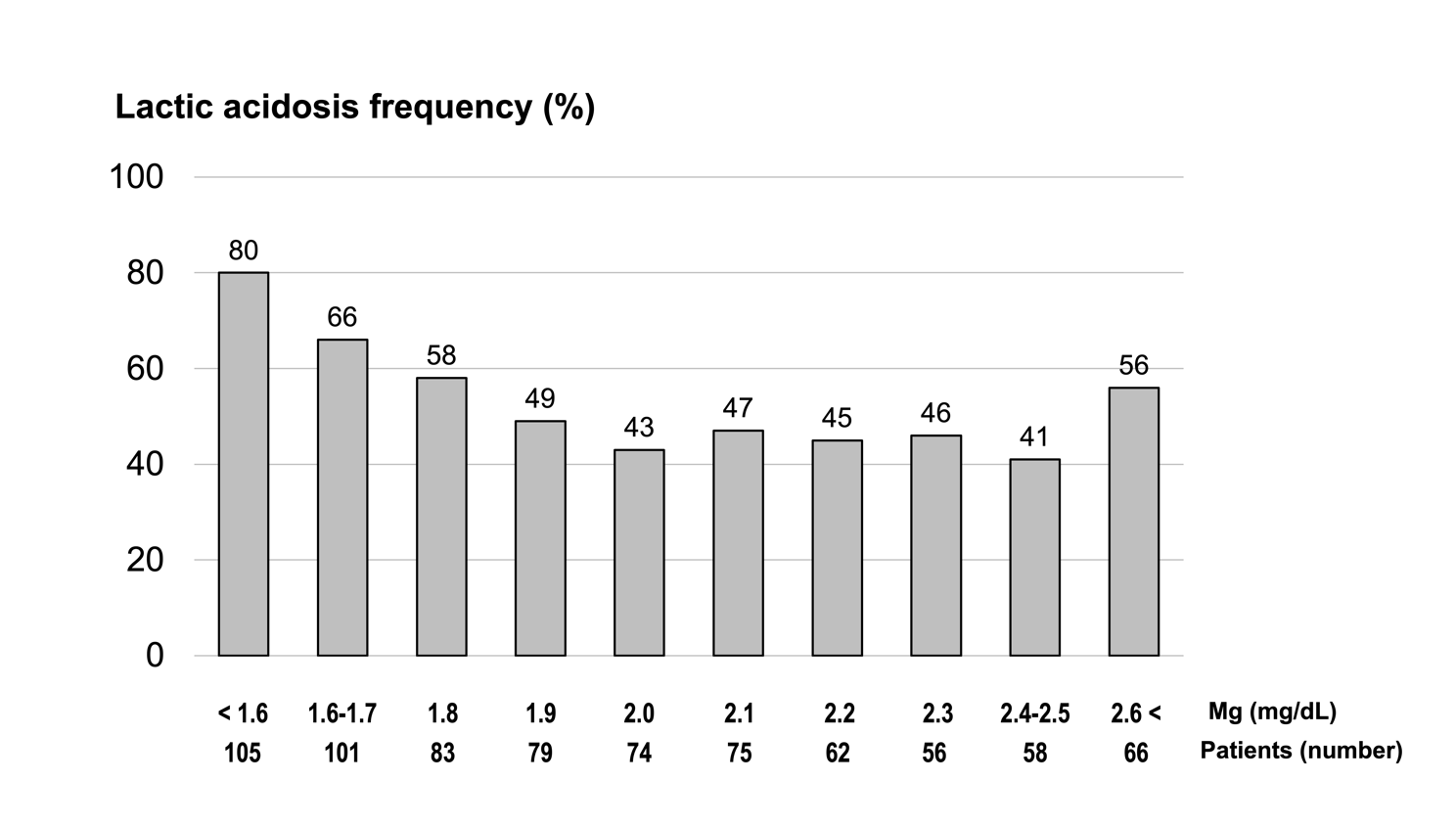
**The frequency of lactic acidosis was sorted into ten equal parts according to serum magnesium concentration on ICU admission. Abbreviations: Mg, magnesium.

**eTable 1. Comparison between Need for vasopressors and non-vasopressors**

|  | Need for vasopressors  N = 558 | Non-vasopressors  N = 201 | *P*-value |
| --- | --- | --- | --- |
| Age, years, median (IQR) | 69 (61–78) | 65 (53–75) | < 0.001 |
| Male sex, n (%) | 306 (54.8) | 114 (56.7) | 0.65 |
| Magnesium, mg/dL | 2.0 (1.7–2.2) | 2.0 (1.8–2.2) | 0.52 |
| Lactate, mmol/L | 2.5 (1.6–4.4) | 1.6 (1.1–2.2) | < 0.001 |
| Lactic acidosis on day1 | 357 (64.0) | 63 (31.3) | < 0.001 |
| Mechanical ventilation | 507 (90.9) | 122 (60.7) | < 0.001 |
| Renal replacement therapy | 170 (30.5) | 12 (6.0) | < 0.001 |
| SOFA score on ICU admission, median (IQR) | 8 (6–11) | 4 (2–6) | < 0.001 |
| APACHE Ⅱ score, median (IQR) | 26 (20–31) | 19 (14–24) | < 0.001 |
| Hospital mortality, n (%) | 115 (20.6) | 16 (8.0) | < 0.001 |

Continuous and categorical data are presented as medians with interquartile ranges (25th and 75th percentiles) and counts with percentages, respectively.

Abbreviations: IQR, interquartile range (first quartile to third quartile); SOFA, Sequential Organ Failure Assessment; APACHE, Acute Physiology and Chronic Health Evaluation.

**eTable 2. Comparison of serum lactate parameters according to serum magnesium levels on ICU admission in subgroup of need for vasopressor**

|  | Hypomagnesemia  (< 1.6 mg/dL) | Normomagnesemia (1.6–2.4 mg/dL) | Hypermagnesemia  (> 2.4 mg/dL) | *P*-value |
| --- | --- | --- | --- | --- |
| On ICU admission | N = 86 | N = 398 | N = 74 |  |
| Lactate, mmol/L | 4.3 (2.8–6.4) | 2.4 (1.5–3.8) ** | 2.3 (1.4–4.7) ** | < 0.001 |
| Lactic acidosis n (%) | 75 (87.2) | 240 (60.3) ** | 42 (56.8) ** | < 0.001 |
| Severe lactic acidosis n (%) | 47 (54.7) | 91 (22.9) ** | 21 (28.4) ** | < 0.001 |

Continuous and categorical data are presented as medians with interquartile ranges (25th and 75th percentiles) and counts with percentages, respectively.

Multiple comparisons were performed using the Steel–Dwass test, chi-square test, or Fisher's exact test with the Bonferroni correction. Comparison with hypomagnesemia: *, P<0.05; **, P<0.01. Comparison with normomagnesemia: †, P<0.05; ††, P<0.01.

Abbreviations: ICU, intensive care unit.

**eTable 3. Comparison of serum lactate parameters according to serum magnesium levels on ICU admission in subgroup of no need for vasopressor**

|  | Hypomagnesemia  (< 1.6 mg/dL) | Normomagnesemia (1.6–2.4 mg/dL) | Hypermagnesemia  (> 2.4 mg/dL) | *P*-value |
| --- | --- | --- | --- | --- |
| On ICU admission | N = 19 | N = 160 | N = 22 |  |
| Lactate, mmol/L | 2.0 (1.2–3.1) | 1.5 (1.0–2.2) | 1.5 (1.1–2.3) | 0.28 |
| Lactic acidosis n (%) | 9 (47.3) | 48 (30.0) | 6 (27.3) | 0.28 |
| Severe lactic acidosis n (%) | 1 (5.3) | 12 (7.5) | 2 (9.1) | 0.90 |

Continuous and categorical data are presented as medians with interquartile ranges (25th and 75th percentiles) and counts with percentages, respectively.

Multiple comparisons were performed using the Steel–Dwass test, chi-square test, or Fisher's exact test with the Bonferroni correction. Comparison with hypomagnesemia: *, P<0.05; **, P<0.01. Comparison with normomagnesemia: †, P<0.05; ††, P<0.01.

Abbreviations: ICU, intensive care unit.
